# Real-World Utilization of the Novavax Adjuvanted Recombinant Protein COVID-19 Vaccine in France During the 2022–2024 Period

**DOI:** 10.3390/vaccines14090790

**Published:** 2026-09-09

**Authors:** Stephanie H. Read, Lucie Kutikova, Gaelle Gusto, Raissa Kapnang, Nadia Quignot, Artak Khachatryan, Clotilde El Guerche-Séblain, Vincent Petit, Jonathan Fix, Matthew D. Rousculp, Cécile Janssen, Odile Launay

**Affiliations:** 1 Certara, London EC2Y 5EB, UK; 2 Novavax, Inc., Gaithersburg, MD 20878, USA; 3 Certara, 75008 Paris, France; 4 Sanofi Vaccines, 69007 Lyon, France; 5 Centre Hospitalier Annecy-Genevois Annecy Site, 74370 Épagny-Metz-Tessy, France; 6 Assistance Publique-Hôpitaux de Paris, Université Paris Cité, 75012 Paris, France

**Keywords:** France, COVID-19 vaccine, vaccination, vaccine utilization, vaccine uptake

## Abstract

Background/Objectives: The Novavax COVID-19 vaccine (Nuvaxovid™) was authorized in France in December 2021, assisting in ending the global pandemic and providing an alternative to other COVID-19 vaccines. Understanding Nuvaxovid recipients’ profile is essential for guiding vaccination decision-making and maintaining protection amid declining COVID-19 vaccination rates. Methods: This retrospective, observational study characterizes the evolution of French Nuvaxovid recipients’ demographic and clinical characteristics using the Système National des Données de Santé (SNDS) database. Five study seasons (spring 2022, autumn 2022, spring 2023, autumn 2023, spring 2024) were defined based on French vaccination guidelines and Nuvaxovid authorization dates. T-test and Chi-square tests were used to compare between consecutive seasons and subgroups. Results: Nuvaxovid administration was reported in 9805 recipients during spring 2022, 5601 during autumn 2022, 531 during spring 2023, 980 during autumn 2023, and 909 during spring 2024. Statistically significant increases in the proportions of high-risk (spring 2022: 35.4%, spring 2024: 90.5%) and ≥65-year-old (spring 2022: 19.9%, spring 2024: 84.4%) Nuvaxovid recipients were observed, *p* < 0.05. Comorbidities, notably cardiovascular disease, became progressively more prevalent (spring 2022: 7.1%, spring 2024: 33.7%, *p* < 0.05). Nuvaxovid was used nationwide, with a higher proportion of recipients in the southeast, a region typically exhibiting lower vaccine coverage. Most Nuvaxovid recipients increasingly received a heterologous regimen (autumn 2022: 91%, spring 2024: 98.6%, *p* < 0.05), with Nuvaxovid as the ≥4th dose (55–97.2%, *p* < 0.05). Meanwhile, higher proportions of <65-year-old and not-at-risk recipients received Nuvaxovid as primary series (autumn 2022: 46.9% <65-year-old vs. 5.6% ≥65-year-old, 53.9% not at risk vs. 11.9% at risk, *p* < 0.05). Conclusions: The use of Nuvaxovid evolved in accordance with changing clinical recommendations, and mix-and-match vaccination strategies and heterologous vaccination patterns suggest that it served as an important protein-based alternative for those switching from other COVID-19 vaccines. Nuvaxovid offers a valuable choice, so that the most vulnerable continue to be vaccinated.

## 1. Introduction

Severe acute respiratory syndrome coronavirus 2 (SARS-CoV-2) continues to circulate globally, requiring ongoing management and preventive measures to protect vulnerable populations [1,2,3,4,5]. Consequently, COVID-19 remains a global health concern with considerable residual risk remaining in elderly or immunocompromised individuals, and in populations with certain comorbidities.

Nuvaxovid™ is a Matrix-M^®^-adjuvanted recombinant SARS-CoV-2 spike glycoprotein vaccine that provides a non-mRNA alternative to other COVID-19 vaccines. Nuvaxovid (ancestral Wuhan strain) received initial marketing authorization from the European Medicines Agency as a two-dose primary series in December 2021 and later as a booster on 12 September 2022. Variant-adapted versions were developed to address new strains of the virus as they emerged, resulting in an XBB.1.5 variant-adapted version authorized in October 2023, a JN.1 variant-adapted version authorized in October 2024, and the XFG variant-adapted version under development for the 2026–2027 vaccination season [6,7,8,9]. Evidence from randomized, placebo-controlled clinical trials has demonstrated that Nuvaxovid provides durable protection when used either as a primary series or as a booster [10,11,12,13,14].

Nuvaxovid has been available in France since January 2022 and is currently recommended by the French ‘Haute Autorité de Santé’ (HAS) as an alternative to mRNA vaccines [15]. As of June 2023, ~80% of the French population completed the first vaccination cycle, and ~60% received at least one booster dose [16].

In France, annual vaccination against COVID-19 is recommended by the Technical Committee on Vaccinations (CTV) for people 65 years of age and older and people less than 65 years old who are at risk of severe COVID-19, with supplementary booster doses in the spring recommended for people who are 80 years and older, immunocompromised, or living in long-term care units [16]. Retrospective evaluations provide key insights for guiding vaccination decision-making and maintaining protection amid declining COVID-19 vaccinations.

Characterizing Nuvaxovid recipients specifically, rather than the overall vaccinated population, is warranted for several reasons. First, Nuvaxovid is the principal Matrix-M-adjuvanted, protein-based alternative available in France to mRNA and adenoviral-vector platforms, and HAS/CTV guidance has specifically positioned it for individuals who decline or have contraindications to mRNA vaccination [15,17,18]; whether recipients in practice align with this intended target group is a distinct, policy-relevant question that aggregate national vaccination statistics cannot answer, since these do not capture the reason underlying vaccine selection. Second, real-world monitoring of individual vaccine products, particularly those authorized under conditional marketing authorization, is a standard component of EU pharmacovigilance and risk-management planning [19], and a comparable single-product characterization has been published for Nuvaxovid in Germany [20]. Third, real-world COVID-19 vaccine uptake in France and the EU during this period occurred against a backdrop of pronounced market concentration around a single mRNA product. The European Commission’s largest vaccine procurement contract, for up to a further 1.8 billion Comirnaty doses, agreed in May 2021, was negotiated directly between the Commission President and the manufacturer. By the end of 2021, the EU had already received almost 952 million vaccine doses, predominantly from Pfizer/BioNTech, sufficient to fully vaccinate 80% of the EU adult population [21], with a further 1.8 billion doses contracted for 2022–2023 alone. Understanding how the profile of this comparatively small group of Nuvaxovid recipients evolved therefore provides insight into how a minority-platform alternative was used in practice as the program matured—offering insights not available from published national-level or mRNA-focused cohort studies [22,23,24,25,26].

As such, the primary objective of this study was to describe Nuvaxovid uptake and track the evolution of sociodemographic and clinical characteristics of Nuvaxovid recipients in France between 2022 and 2024.

## 2. Materials and Methods

### 2.1. Study Design and Population

This retrospective, observational cohort study characterized Nuvaxovid recipients in France using data from the Système National des Données de Santé (SNDS), a comprehensive national administrative database encompassing health information for over 99% of the French population. The SNDS integrates longitudinal claims data from inpatient and outpatient encounters by linking three principal sources: the national hospital discharge database (Programme de Médicalisation des Systèmes d’Information, PMSI), the national health insurance claims database (Système National d’Information Inter-Régimes de l’Assurance Maladie, SNIIRAM), and the national death registry. In addition, the SNDS was linked to two COVID-19 specific databases, Vaccin COVID-19 (VAC-SI) and Système d’Informations de DEPistage (SI-DEP), which include vaccination and infection status.

**Figure 1:**
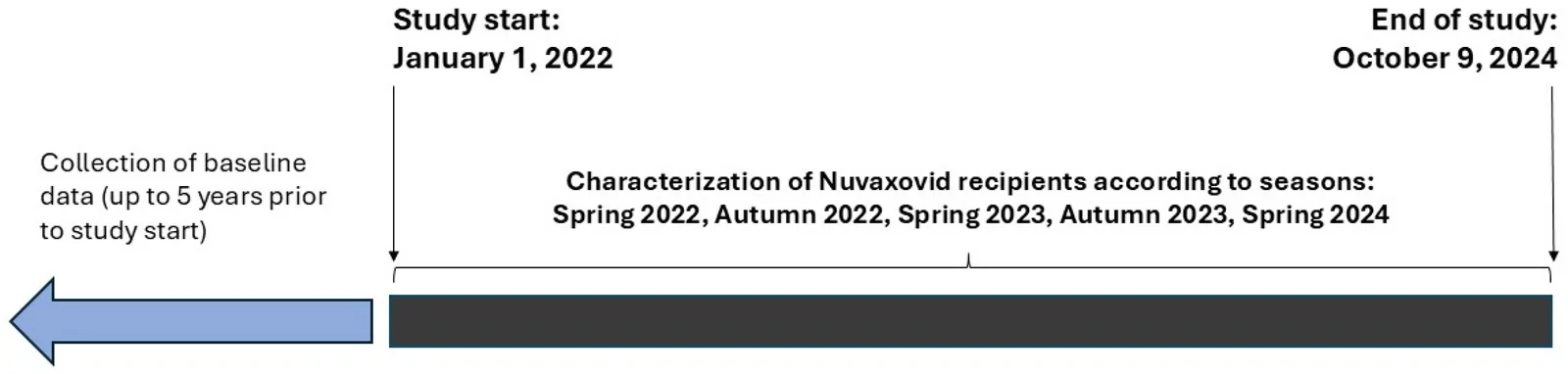
Study design.

The study population comprised recipients of at least one Nuvaxovid dose between 1 January 2022 and 9 October 2024 (Figure 1), reflecting the marketing authorization date for the original vaccine and the end of the authorization period for the XBB.1.5 variant-adapted version, respectively. Nuvaxovid recipients were identified using Code d’Identification de Presentation (CIP) and Unité Commune de Dispensation (UCD) codes [27]. Because CIP codes are not dose- and variant adaptation-specific, the inclusion criteria to the study considered the timing of age and market authorization of variant adaptations [7,8].

To be included in the final analytic dataset, eligible vaccinees must have received at least a second dose of a two-dose primary series or a booster dose of the Nuvaxovid vaccine during the study period, had plausible data (e.g., death date, if any, occurring after vaccination date, gender, birth date, at least one consultation date with a practitioner or at hospital), and were aligned with the approved indication (Supplemental Table S1). A primary series was defined as two doses of Nuvaxovid, administered 18 to 56 days apart, without prior receipt of a COVID-19 vaccine [17,19]. As Nuvaxovid was not approved for use as a booster until 12 September 2022, all Nuvaxovid recipients during the spring 2022 season with a previous record of a COVID-19 vaccination were excluded. Booster doses were defined as receipt of at least one dose with prior Nuvaxovid or other COVID-19 vaccine, administered at least 90 days apart from previous COVID-19 vaccine [15,17,18,19]. Recipients were categorized within five seasons, spring 2022, autumn 2022, spring 2023, autumn 2023, and spring 2024, as defined based on French vaccination guidelines and Nuvaxovid market authorization dates (Table 1). An individual’s index date was defined as the date of administration of Nuvaxovid in the respective season; where recipients had multiple Nuvaxovid vaccines during the season, the last administered dose was the index date.

### 2.2. Outcomes

Sociodemographic and clinical characteristics evaluated included age, gender, geographical region, at-risk status, comorbidities, Nuvaxovid regimen type and schedule, influenza vaccine use, and socio-professional categories. Social deprivation was measured using the French Deprivation Index (FDEP), a geographical social deprivation index based upon several sociodemographic domains (unemployment rate, labor rate, rate of high school graduates, median income per household, status of universal complementary health insurance delivered), and operationalized in quintiles [32]. Recipients at risk of severe disease, defined according to the CTV in France, [27] included adults ≥ 65 years of age, individuals ≥ 12 years of age with specific comorbidities (complicated hypertension, cardiac, vascular, hepatic, renal, pulmonary pathologies, diabetes, obesity, cancers, transplant recipients, trisomy 21, psychiatric disorders or dementia), immunocompromised individuals, and nursing home residents.

Where possible, algorithms specified in the Cartographie des Pathologies et des Dépenses (mapping of diseases and expenditures) were used to inform the International Classification of Disease, version 10 (ICD-10) codes to define the comorbidities [33]. These algorithms were developed from the Datamart de Consommation Inter-Regimes from SNIIRAM and the PMSI to enable the identification of diseases in the SNDS in a given year by means of medical algorithms. In the absence of an available Cartography algorithm, code lists were obtained from COVID-19 publications, which used the SNDS database [5,25].

### 2.3. Statistical Analysis

Descriptive statistics such as frequency, means and medians were used to summarize Nuvaxovid recipient characteristics by season. *t*-tests for continuous and Chi-square tests for categorical variables were used to identify statistically significant differences between consecutive seasons as well as between subgroups. Subgroup analyses were conducted according to age categories (i.e., <65 vs. ≥65 years and <80 vs. ≥80 years), classification of those at risk of severe disease versus those not at risk, and ≥12year-old participants with comorbidities versus those without comorbidities.

All analyses were conducted using SAS^®^ Statistical Package, version 9.4 (SAS Institute, Cary, NC, USA). This study was conducted in compliance with the French National Commission for Information Technology and Freedoms (Commission Nationale de l’Informatique et des Libertés (CNIL)) MR-008 methodology (Méthodologie de Référence 008) for processing personal health data from the SNDS [34].

## 3. Results

### 3.1. Recipient Characteristics

Nuvaxovid was administered to 9805 recipients in spring 2022, 5601 in autumn 2022, 531 in spring 2023, 980 in autumn 2023, and 909 in spring 2024 (Table 2). The mean age of Nuvaxovid recipients increased from 49 years in spring 2022 to 78 years in spring 2024; by the spring of 2024, most recipients (84%) were ≥65 years of age, up from 20%, *p* < 0.05. Nuvaxovid was used across France, with a higher proportion of recipients living in the southeast. Most recipients resided in urban areas, and the proportions were similar across seasons from spring 2022 (68%) to spring 2024 (64.1%), with the lowest proportion during spring 2023 (58.4%). The proportion of recipients who were private-sector workers or self-employed significantly decreased from 53.7% in spring 2022 to 8.6% in spring 2024, while the proportion of retirees increased from 16.5% to 63.4%, *p* < 0.05. The proportion of recipients who resided in the least deprived areas in France varied over time, declining from 36.7% in spring 2022 to 28.7% in spring 2023, before increasing to 39.7% by spring 2024. Less than 10% of Nuvaxovid recipients were medical aid beneficiaries among all seasons.

**Figure 2:**
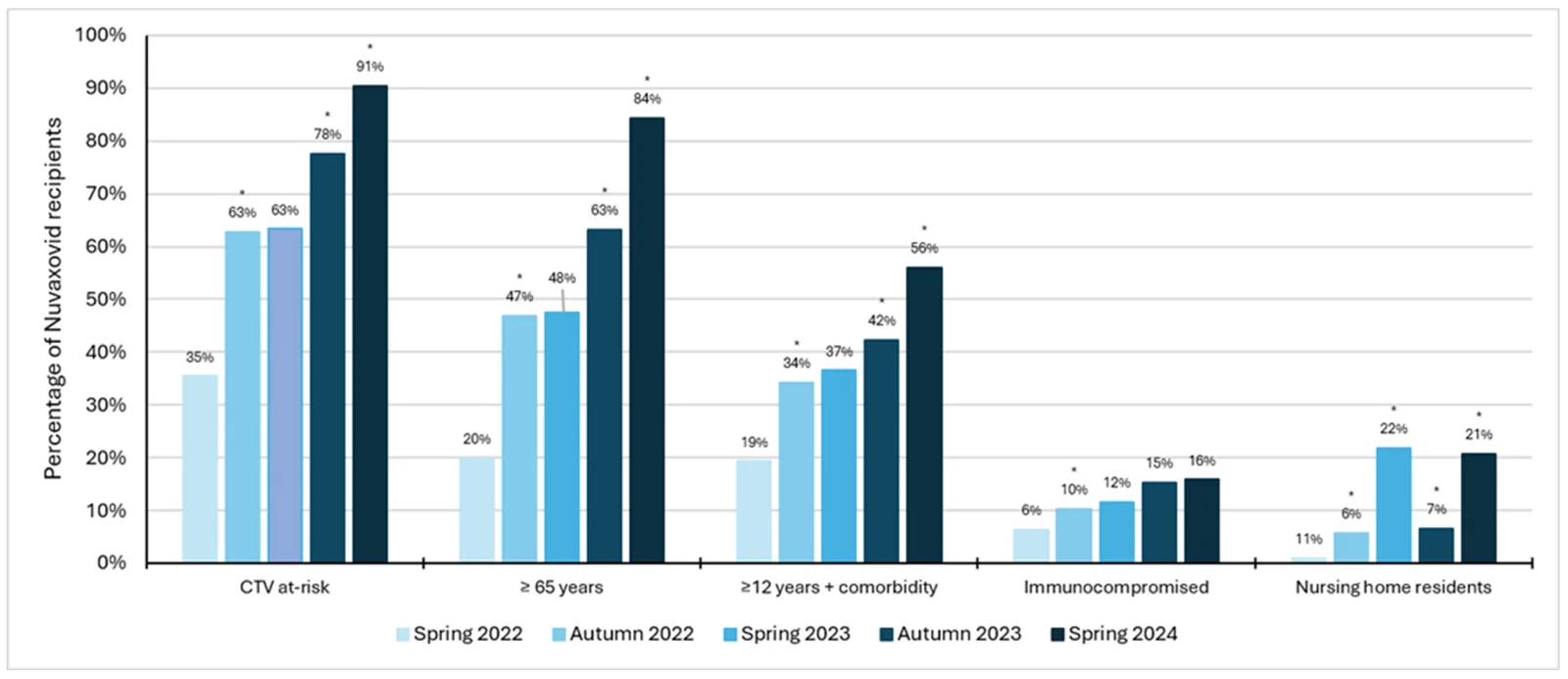
Distribution of Nuvaxovid recipients by CTV high-risk groups. * *p*-value < 0.05 versus previous season, CTV: Technical Committee on Vaccinations.

The frequency of participants with a CTV designation of at risk of severe disease [27] increased significantly over time from 35% in spring 2022 to 91% in spring 2024, *p* < 0.05 (Figure 2), as did the proportion with two or more comorbidities (6% to 28%, *p* < 0.05) and more than three comorbidities (2% to 15%, *p* < 0.05). Similar increases were observed from spring 2022 to spring 2024 in the proportions of the most commonly reported comorbidities: cardiovascular disease (7% to 34%, *p* < 0.05), diabetes (5% to 19%, *p* < 0.05), pulmonary disease (4% to 12%, *p* < 0.05), and psychiatric disorders (4% to 11%, *p* < 0.05) (Supplemental Figure S1).

From autumn 2022 onwards, the majority received the vaccine as a booster, with the proportion rising from 73% in autumn 2022 to 99.8% by spring 2024, *p* < 0.05 (Table 3); during that period, a consistently high proportion of booster doses were heterologous in nature, rising from 91% to 98.6%. In spring 2024, 97.2% of recipients had Nuvaxovid as their fourth or more COVID-19 dose, a significant increase from 55% in August 2022, *p* < 0.05. Most recipients received their vaccine at a physician office or pharmacy, with frequencies increasing from 57.1% in spring 2022 to 91.5% in autumn 2023. Throughout the study period, a majority were administered their vaccine by a pharmacist (as high as 80.5% in autumn 2023). Concomitant administration of the influenza vaccine occurred only during the autumn seasons and was infrequent (3.3% in autumn 2022 and 3.4% in autumn 2023).

### 3.2. Subgroup Analyses

Differences were noted among characteristics of Nuvaxovid recipients stratified by age. In autumn 2022, Nuvaxovid was administered as a primary series more often to younger recipients (<65 years, 46.9%) than it was to older recipients (≥65 years, 5.6%), *p* < 0.05; a similar pattern was seen for recipients < 80 years of age versus those ≥80 years of age (Table 4). In the same time frame, a greater proportion of older (≥65 years) than younger (<65 years) recipients (80.5% vs. 32.5% respectively, *p* < 0.05) received Nuvaxovid as their fourth or more COVID-19 vaccine dose. In terms of their comorbidities, older Nuvaxovid recipients (≥65 years) were more likely than younger recipients (<65 years) to have at least one comorbidity (47.4% vs. 22.6%, respectively, *p* < 0.05).

When stratified by CTV-defined risk status for severe disease, a higher proportion of CTV not-at-risk recipients received Nuvaxovid as the primary series (53.9%) than did recipients who were at risk (11.9%), *p* < 0.05 (Table 4). In addition, at-risk Nuvaxovid recipients were older on average than not-at-risk recipients (mean age 69 years vs. 42 years, *p* < 0.05) and in autumn 2022 (as well as in spring 2024), a greater proportion of at-risk recipients than those not at risk received Nuvaxovid as their fourth or more COVID-19 vaccine dose (72.2% vs. 26%, respectively, *p* < 0.05).

Additional subgroup analyses conducted between strata of age (<80 vs. ≥80 years) and recorded presence of comorbidities (≥12 years old with comorbidities versus without comorbidities) revealed similar patterns (Supplemental Table S2).

## 4. Discussion

This retrospective study presents important insights into the evolution of the sociodemographic and clinical profiles of individuals who received Nuvaxovid. The study used a large, nationwide database covering approximately 99% of the French population. The SNDS provided comprehensive, longitudinal data on vaccine recipients, allowing for a detailed characterization of individuals who received Nuvaxovid in France. As a result, the study population is likely to be representative of Nuvaxovid recipients nationally.

The majority (>95%) of individuals received Nuvaxovid as a heterologous booster regimen, which may reflect the administration of mix-and-match vaccination strategy in France [13,35]. Evidence from clinical trials has demonstrated that administering different COVID-19 vaccine technology platforms, including Nuvaxovid, in a heterologous sequence can enhance immune protection [13,24,36]. Specifically, this strategy increased antibody titters and broadened neutralizing activity against SARS-CoV-2 variants, thereby offering greater and more durable protection compared to homologous booster regimens. Additionally in 2022, 87.7% of all COVID-19 recipients in France had previously received homologous mRNA vaccines [24]. Despite lower overall Nuvaxovid uptake compared to mRNA vaccines, heterologous vaccination patterns suggest Nuvaxovid may provide an important protein-based alternative for specific populations transitioning from mRNA platforms.

Nuvaxovid was administered across France, with the highest proportions of recipients located in the southeast region of France, a region typically associated with lower vaccine coverage [37,38]. Vaccine hesitancy poses a considerable barrier to the effectiveness of COVID-19 vaccination efforts [39,40,41]. As public attitudes towards COVID-19 vaccines evolve, broadening choice with diverse vaccine platforms can enhance vaccination rates and bolster protection of vulnerable populations.

The comparatively small number of Nuvaxovid recipients relative to the broader French vaccinated population reflects, in part, the scale on which mRNA vaccines were procured and deployed across the EU from the outset of the COVID-19 vaccination campaign, including a May 2021 European Commission contract reserving up to a further 1.8 billion Comirnaty doses [42]. Real-world uptake mirrored this concentration: among French recipients ≥ 75 years of age vaccinated by February 2021, 92% received Comirnaty [43], and in a nationwide cohort of over 11 million adults ≥ 50 years of age vaccinated by April 2021, 63.6% received BNT162b2, 28.8% ChAdOx1-S, and 7.6% mRNA-1273 [22]. As adenoviral-vector vaccines were progressively restricted from mid-2021 following safety signals, and as Nuvaxovid was not authorized in the EU until December 2021, non-mRNA options remained a minority pathway throughout the study period and used predominantly by the subset of patients with contraindications to, or reluctance toward, mRNA vaccines, consistent with HAS guidance positioning Nuvaxovid specifically as an mRNA alternative [15,17,27]. By the 2024–2025 season, following discontinuation of Nuvaxovid supply in France, mRNA (JN.1-adapted) was the sole vaccine used for COVID-19 boosters nationally, underscoring that the alternative-platform pathway this study characterizes was time-limited rather than an enduring feature of the French immunization landscape.

In spring 2022, a substantial number of individuals received Nuvaxovid as part of their primary series (*N* = 9805). Compared with recipients of other COVID-19 vaccines administered as primary series earlier in the pandemic in spring and summer 2021, Nuvaxovid recipients were younger than reported recipients of other COVID-19 vaccines (49 years vs. 57 years, respectively) [26]. Over time, Nuvaxovid use transitioned towards older age groups and retired individuals, coinciding with changes to French clinical guidelines. Globally, shifts in recommendations towards higher risk groups have been consistently observed [44]. The mean age of recipients in this study increased from 49 years in spring 2022, when all doses were administered as part of the primary series, to 78 years by spring 2024, when Nuvaxovid was used primarily as a booster. By spring 2024, 84.4% of recipients were ≥65 years old, demonstrating the targeting of older adults from autumn 2022 onwards. These findings are consistent with 2022 data from Germany, where recipients of Nuvaxovid primary series were, on average, 51 years old compared to those receiving Nuvaxovid as a booster (71 years old) [20].

Consistent with the change in use of Nuvaxovid towards older age groups over time, Nuvaxovid recipients increasingly comprised individuals at greater risk of severe COVID-19. The proportion of recipients with at least one comorbidity rose from 19.3% in spring 2022 to 56.1% in spring 2024. Compared with a reported study of French recipients of other COVID-19 vaccines in 2021, Nuvaxovid primary series recipients in spring 2022 were less likely to have cardiovascular disease (7% vs. ~17.9%), diabetes (4.9% vs. 9.5%) or pulmonary disorders (4% vs. 6.7%) [26]. In the general population in France in 2019, the prevalence of cardiovascular disease, diabetes and pulmonary disease was 21%, 6% and 5.5%, respectively [45]. By spring 2024, the most common comorbidities were cardiovascular disease (33.7%), diabetes (19%), pulmonary disease (11.7%), and psychiatric disorders (11.2%). While the increased frequency of administration to vulnerable populations may have been driven by evolving recommendations, Nuvaxovid may play a role in addressing an unmet need within these groups.

The study has several limitations. Only data on Nuvaxovid recipients were available for analysis, restricting the ability to compare characteristics with those who either did not receive a COVID-19 vaccine or received an mRNA COVID-19 vaccine during a particular season. Additionally, the data were primarily collected for administrative purposes rather than for research, which may have resulted in misreporting, misclassification, or missing information. In particular, the identification of comorbidities relied on hospital diagnoses and records, which may have led to an underestimation of conditions that do not result in hospitalization or reimbursement. For example, the lack of body mass index data in the database may have resulted in underreporting of obesity prevalence. To minimize the impact of this limitation, established and validated algorithms were applied where available. Additionally, as a result, it was not possible to comment on relevant differences between populations who received different types of vaccines, nor was it possible to evaluate the distributions of demographic and clinical characteristics among the overall source population and those recommended to be vaccinated in a given season. Because national vaccination recommendations, overall COVID-19 vaccine uptake, the availability and use of competing vaccines, and public acceptance of vaccination all evolved concurrently over the study period, this single-arm design cannot determine whether the observed temporal changes in Nuvaxovid recipient characteristics reflect genuine shifts in who selected Nuvaxovid or instead reflect these broader, concurrent changes. Patient preference for Nuvaxovid versus an mRNA vaccine was also not captured. Accordingly, the findings describe the evolving characteristics of Nuvaxovid recipients over time, rather than the utilization of Nuvaxovid relative to other available COVID-19 vaccines, and should be interpreted as such. Large national cohort studies using the same SNDS/VAC-SI data infrastructure have already established the comparative effectiveness and safety of mRNA and adenoviral-vector vaccines against unvaccinated controls in France [5,23,24,25,26,43], providing complementary context.

## 5. Conclusions

Over time, the use of Nuvaxovid evolved i coinciding with changing clinical recommendations, and transitioning towards older adults and vulnerable at-risk individuals. Patterns of heterologous vaccination suggest that Nuvaxovid may serve an important role as an alternative for those switching from other COVID-19 vaccines. In the context of declining vaccination rates, Nuvaxovid remains a valuable choice, so that the most vulnerable continue to be vaccinated.

## Figures and Tables

**Table 1:** Definition of study seasons.

Season	Start Date	Finish Date	Recommended Population
Spring 2022 Primary series [6]	1 January 2022 (MA 20 December 2021) [6]	11 September 2022 [7]	Nuvaxovid recommended to ≥18-year-olds who refuse mRNAs or contraindicated to mRNAs [19,20]
Autumn 2022 [7]	12 September 2022 [7]	26 April 2023 [28]	Residents of nursing homes, ≥60 years old, immunocompromised, people with comorbidities at risk of severe COVID-19, pregnant, people living in proximity to vulnerable [15,29]
Spring 2023 [19]	27 April 2023 [28]	30 October 2023 [8]	≥80 years old, immunocompromised, residents of nursing homes, people at very high risk of severe illness [28]
Autumn 2023 XBB-adapted [20]	31 October 2023 [8]	14 April 2024 [29]	≥65 years old, immunocompromised, people ≥ 12 years with comorbidities at risk of severe COVID-19, pregnant, residents of nursing homes, people living in proximity to vulnerable [30]
Spring 2024 [29]	15 April 2024 [31]	9 October 2024 [9]	≥80 years old, immunocompromised, residents of nursing homes, people at very high risk of severe illness [31]

MA: market authorization.

**Table 2:** Demographic characteristics of Nuvaxovid recipients.

	Spring 2022 N (%)	Autumn 2022 N (%)	Spring 2023 N (%)	Autumn 2023 N (%)	Spring 2024 N (%)
**Total recipients**	9805 (100)	5601 (100)	531 (100)	980 (100)	909 (100)
**Age**					
** Mean (SD)**	49 (17)	59 (20) *	58 (22)	67 (16) *	78 (15) *
**Gender**					
** Male**	4418 (45.1)	2610 (46.6)	241 (45.4)	453 (46.2)	439 (48.3)
** Female**	5387 (54.9)	2991 (53.4)	290 (54.6)	527 (53.8)	470 (51.7)
**Geographic location of vaccination ^a, b^**					
** Île-de-France**	1659 (18.2)	857 (18.8)	39 (17.8)	86 (15.2)	120 (20.9) *
** Northeast**	1543 (16.9)	722 (15.8)	38 (17.4)	93 (16.4)	49 (8.5) *
** Southeast**	1988 (21.8)	1293 (28.3) *	65 (29.7)	149 (26.3)	128 (22.3)
** Southwest**	1750 (19.2)	852 (18.7)	37 (16.9)	124 (21.9)	64 (11.1) *
** East**	1410 (15.4)	734 (16.1)	34 (15.5)	113 (20.0)	209 (36.3) *
** Overseas territories**	782 (8.6)	109 (2.4) *	<10 (<1)	<10 (<1) *	<10 (<1)
**Residence ^b^**					
** Rural**	1800 (32.0)	1136 (32.9)	123 (41.6) *	263 (40.0)	200 (35.9)
** Urban**	3826 (68.0)	2313 (67.1)	173 (58.4) *	394 (60.0)	357 (64.1)
**Socio-professional category ^b^**					
** Healthcare**	109 (1.1)	92 (1.7) *	12 (2.3)	13 (1.3)	<10 (<1) *
** State**	713 (7.4)	234 (4.2) *	30 (5.7)	46 (4.7)	19 (2.2) *
** Private sector or self-employed**	5186 (53.7)	1820 (32.6) *	146 (27.5) *	185 (18.9) *	75 (8.6) *
** Retiree**	1591 (16.5)	2094 (37.6) *	184 (34.7)	484 (49.5) *	550 (63.4) *
**Social French Deprivation Index (FDEP) ^b^**					
** Least deprived Q1–Q2**	2049 (36.7)	1239 (33.1) *	94 (28.7)	278 (38.1) *	252 (39.7)
** Q3**	1859 (33.3)	1100 (29.4) *	102 (31.1)	206 (28.2)	155 (24.4)
** Most Deprived Q4–Q5**	1673 (30.0)	1404 (37.5) *	132 (40.2) *	246 (33.7) *	228 (35.9)
**Beneficiaries of medical aid**					
** Yes (~lower income)**	871 (8.9)	401 (7.2) *	52 (9.8) *	42 (4.3) *	59 (6.5) *
** No**	8934 (91.1)	5200 (92.8) *	479 (90.2) *	938 (95.7) *	850 (93.5) *
**Practice/insurance type ^b^**					
** Pre-approved physicians (~lower income)**	4126 (70.2)	2596 (70.6)	232 (73.9)	467 (66.8) *	377 (66.6)
** Fee-for-service physicians**	1755 (29.8)	1083 (29.4)	82 (26.1)	232 (33.2) *	189 (33.4)

SD: standard deviation, FDEP: French Deprivation Index. * *p* < 0.05 versus previous season. ^a^ Île-de-France, Northeast (Grand Est, Hauts-de-France, Bourgogne-Franche-Comté), Southeast (Auvergne-Rhône-Alpes, Provence-Alpes-Côte d’Azur, Corse), Southwest (Nouvelle-Aquitaine, Occitanie), West (Bretagne, Pays de la Loire, Centre-Val de Loire, Normandie), Overseas Territories (Guadeloupe, Martinique, Guyane, La Réunion, Mayotte). ^b^ Limitation: a proportion of data were missing.

**Table 3:** Vaccine utilization of Nuvaxovid recipients.

	Spring 2022 N (%)	Autumn 2022 N (%)	Spring 2023 N (%)	Autumn 2023 N (%)	Spring 2024 N (%)
**Total recipients**	9805 (100)	5601 (100)	531 (100)	980 (100)	909 (100)
**Nuvaxovid Schedule**					
Primary Series	9805 (100.0)	1543 (27.5) *	109 (20.5) *	12 (1.2) *	<10 (<1.0) *
Booster	0 (0.0)	4091 (73.0) *	422 (79.5) *	968 (98.8) *	907 (99.8) *
**Regimen Type**					
Homologous booster	0 (0.0)	369 (9.0)	73 (17.3) *	109 (11.3) *	13 (1.4) *
Heterologous booster	0 (0.0)	3722 (91.0)	349 (82.7) *	859 (88.7) *	894 (98.6) *
**Current COVID-19 vaccine dose ^a^**					
<4	9805 (100.0)	2520 (45.1) *	225 (42.3)	157 (16.0) *	26 (2.9) *
≥4	0 (0.0)	3081 (55.0) *	306 (57.6)	823 (83.9) *	883 (97.2) *
**COVID-19 vaccine prior to index date**					
None	9805 (100.0)	1510 (27.0)	109 (20.5) *	12 (1.2) *	<10 (<1.0) *
Prior COVID-19 vaccine	0 (0.0)	4091 (73.0)	422 (79.5) *	968 (98.8) *	907 (99.8) *
Nuvaxovid™	0 (0.0)	502 (9.0)	124 (23.4) *	212 (21.6)	59 (6.5) *
Comirnaty^®^	0 (0.0)	3064 (54.7)	297 (55.9)	759 (77.4) *	858 (94.4) *
Spikevax™	0 (0.0)	1259 (22.5)	116 (21.8)	279 (28.5) *	321 (35.3) *
Jcovden	0 (0.0)	226 (4.0)	28 (5.3	41 (4.2)	14 (1.5) *
Vaxzevria™	0 (0.0)	882 (15.7)	62 (11.7) *	178 (18.2) *	104 (11.4) *
VidPrevtyn Beta™	0 (0.0)	0 (0.0)	12 (2.3) *	87 (8.9) *	109 (12.0) *
**Use of influenza vaccine**					
Concomitant with Nuvaxovid	N/A	191 (3.4)	N/A	32 (3.3)	N/A
**Administration setting**					
Physician office or pharmacy	5599 (57.1)	4873 (87.0) *	420 (79.1) *	897 (91.5) *	694 (76.3) *
Vaccine center	3771 (38.5)	149 (2.7) *	<10 (<1.0) *	<10 (<1.0)	<10 (<1.0)
Hospital	157 (1.6)	16 (0.3) *	<10 (<1.0)	<10 (<1.0)	0 (0.0)
Nursing home	<10 (<1.0)	128 (2.3) *	84 (15.8) *	18 (1.8) *	148 (16.3) *
Other	270 (2.8)	435 (7.8) *	23 (4.3) *	53 (5.4)	62 (6.8)
**Administering healthcare professional ^b^**					
Physician	1166 (20.4)	1454 (29.9) *	172 (38.9) *	103 (12.7) *	51 (7.6) *
Pharmacist	3692 (64.6)	2992 (61.6) *	248 (56.1) *	654 (80.5) *	521 (78.0)
Nurse	772 (13.5)	403 (8.3) *	20 (4.5) *	52 (6.4)	96 (14.4) *
Other	87 (1.5)	11 (0.2) *	<10 (<1.0)	<10 (<1.0)	0 (0.0)

* *p*-value < 0.05 vs. previous season, ^a^ For primary series, two doses are considered as one, ^b^ Limitation: proportion of missing data, NA: not applicable.

**Table 4:** Key characteristics of Nuvaxovid recipients in the autumn 2022 and spring 2024 seasons, by subgroups.

	Autumn 2022, N (%)		Spring 2024, N (%)		Autumn 2022, N (%)		Spring 2024, N (%)	
	<65 Years	≥65 Years	<65 Years	≥65 Years	At Risk	Not At Risk	At Risk	Not At Risk
**Total recipients**	2977 (100)	2624 (100)	142 (100)	767 (100)	3516 (100)	2085 (100)	823 (100)	86 (100)
**Age**								
Mean age (SD), years	45 (15)	76 (7) *	48 (13)	83 (8) *	69 (14)	42 (15) *	81 (11)	47 (14) *
**CTV at risk**								
Overall at risk	892 (30.0)	2624 (100) *	56 (39.4)	767 (100) *	3516 (100)	NA	823 (100)	NA
≥65 years	0 (0.0)	2624 (100) *	0 (0.0)	767 (100) *	2624 (74.6)	NA	767 (93.2)	NA
≥12 years + comorbidity	674 (22.6)	1243 (47.4) *	43 (30.3)	466 (60.8) *	1917 (54.5)	NA	509 (61.8)	NA
Immunocompromised	252 (8.5)	317 (12.1) *	26 (18.3)	118 (15.4)	569 (16.2)	NA	144 (17.5)	NA
Nursing home resident	139 (4.7)	183 (7.0) *	<10 (<1.0)	178 (23.2) *	322 (9.2)	NA	187 (22.7)	NA
**Nuvaxovid regimen type**								
Homologous	216 (13.4)	153 (6.2) *	10 (7.1)	<10 (<1.0) *	216 (6.9)	153 (15.6) *	<10 (<1.0)	<10 (<1.0) *
Heterologous	1392 (86.6)	2330 (93.8) *	130 (92.9)	764 (99.6) *	2894 (93.1)	828 (84.4) *	815 (99.1)	79 (92.5) *
**Nuvaxovid schedule**								
Primary series	1396 (46.9)	147 (5.6) *	<10 (<1.0)	0 (0.0) *	419 (11.9)	1124 (53.9) *	<10 (<1.0)	<10 (<1.0)
Booster	1608 (54.0)	2483 (94.6) *	140 (98.6)	767 (100) *	3110 (88.5)	981 (47.1) *	822 (99.9)	85 (98.9)
**Current COVID-19 vaccine dose ^a^**								
<4	2009 (67.5)	511 (19.5) *	18 (12.7)	<10 (<1.0) *	978 (27.8)	1542 (74.0) *	14 (1.7)	12 (14.0) *
≥4	968 (32.5)	2113 (80.5) *	124 (87.3)	759 (99.0) *	2538 (72.2)	543 (26.0) *	809 (98.3)	74 (86.0) *
**Use of influenza vaccine**								
Concomitant with Nuvaxovid	34 (1.1)	160 (6.1) *	NA	NA	178 (5.1)	13 (0.6) *	NA	NA

* *p*-value < 0.05 subgroup differences, ^a^ For primary series, two doses are considered as one, CTV: Technical Committee on Vaccinations, NA: Not applicable.

## Data Availability

Restrictions apply to the availability of these data. Data were obtained from the Caisse nationale de l’assurance maladie (Cnam). These data are not publicly available due to French data protection regulations. Access is subject to approval by the Health Data Hub and the CNIL. Eligible researchers may submit a request for access via the Health Data Hub portal (https://www.health-data-hub.fr).

## Supplementary Materials

The following supporting information can be downloaded at https://www.mdpi.com/article/10.3390/vaccines14090790/s1, Figure S1: Distribution of Nuvaxovid recipients by CTV comorbidities; Table S1: Attrition of Nuvaxovid recipients; Table S2: Key characteristics of Nuvaxovid recipients by CTV risk and comorbidity subgroups.

## Abbreviations

The following abbreviations are used in this manuscript:

CIP	Code d’Identification de Presentation
CNIL	Commission Nationale de l’Informatique et des Libertés
COVID-19	Coronavirus Disease 2019
CTV	Technical Committee on Vaccinations
ICD-10	International Classification of Disease, version 10
MA	Market Authorization
MR-008	Méthodologie de Référence 008
mRNA	Messenger Ribonucleic Acid
PMSI	Programme de Médicalisation des Systèmes d’Information
SARS-CoV-2	Severe Acute Respiratory Syndrome Coronavirus 2
SI-DEP	Système d’Informations de DEPistage
SNDS	Système National des Données de Santé
SNIIRAM	Système National d’Information Inter-Régimes de l’Assurance Mala
UCD	Unité Commune de Dispensation
VAC-SI	Vacin COVID-19
