# Supplementary material for: Real-World Utilization of the Novavax Adjuvanted Recombinant Protein COVID-19 Vaccine in France During the 2022–2024 Period
Source: Vaccines (Basel). 2026 Sep 9;14(9):790. doi: 10.3390/vaccines14090790 (PMC13611775; doi:10.3390/vaccines14090790)
Supplement: Supplementary file 1 [file vaccines-14-00790-s001.zip › vaccines-4440883-supplementary.pdf]

Supplement

Supplemental Table S1. Attrition of Nuvaxovid Recipients.

	Spring 2022<br>N (%)	Autumn 2022<br>N (%)	Spring 2023<br>N (%)	Autumn 2023<br>N (%)	Spring 2024<br>N (%)
Nuvaxovid recipients with at least 1 dose	19721 (100)	7011 (100)	653 (100)	1018 (100)	934 (100)
Nuvaxovid recipients with plausible data *	14,605 (74.1)	6250 (89.2)	591 (90.5)	998 (98)	918 (98.3)
Received ≥ 2 Nuvaxovid doses and no prior COVID-19 vaccine, or 1 Nuvaxovid dose and prior COVID-19 vaccine	14,579 (73.9)	6244 (89.1)	590 (90.4)	998 (98)	918 (98.3)
Aligned with approved indication **	103,96 (52.7)	6171 (88)	588 (90.1)	994 (97.6)	917 (98.2)
**Primary series:** time between doses 18-56 days apart	9805 (49.7)	1543 (22)	109 (16.7)	12 (1.2)	<10 (<1.0)
**Boosters:** time between doses 90 days apart	0 (0)	4091 (58.4)	422 (64.6)	968 (95.1)	907 (97.1)
Number of Nuvaxovid vaccinations ***	9805 (49.7)	5634 (80.4)	531 (81.3)	980 (96.3)	909 (97.3)
**Number of Nuvaxovid recipients ******	**9805 (49.7)**	**5601 (79.9)**	**531 (81.3)**	**980 (96.3)**	**909 (96.3)**

* Plausible data was defined as assessment of death date, if any, occurring after vaccination date, gender, birth date, or at least one consultation date with a practitioner or at the hospital.

** Nuvaxovid was only approved as a primary series during the Spring 2022 season. As such, patients with a previous record of a COVID-19 vaccination were excluded to align with the primary series definition.

*** Sum of primary series and boosters.

**** Among recipients with multiple vaccines during the same season, the last vaccine was selected for the study/index date.

Supplemental Table S2. Key Characteristics of Nuvaxovid Recipients by CTV Risk and Comorbidity Subgroups.

	Autumn 2022, N (%)		Spring 2024, N (%)		Autumn 2022, N (%)		Spring 2024, N (%)	
	< 80 years	≥ 80 years	< 80 years	≥ 80 years	≥ 12 years + comorbidity	≥ 12 years + no comorbidity	≥ 12 years + comorbidity	≥ 12 years + no comorbidity
**Total recipients**	4895 (100)	706 (100)	344 (100)	565 (100)	1917 (100)	3684 (100)	509 (100)	400 (100)
**Age**								
Mean age (SD), years	48 (16)	86 (4)*	63 (15)	87 (5)*	68 (15)	55 (20)*	81 (12)	73 (18)*
**CTV At-Risk**								
Overall At-risk	3241 (33.9)	706 (100)*	258 (75.0)	565 (100)*	1917 (100)	1599 (43.4)*	509 (100)	314 (78.5)*
≥ 65 years	1713 (17.9)	706 (100)*	202 (58.7)	565 (100)*	1243 (64.8)	1381 (37.5)*	466 (91.6)	301 (75.3)*
≥12 years + comorbidity	1794 (18.7)	413 (58.5)*	150 (43.6)	359 (63.5)*	1917 (100)	NA	509 (100)	NA
Immunocompromised	600 (6.3)	75 (10.6)	65 (18.9)	79 (14.0)	357 (18.6)	212 (5.8)*	119 (23.4)	25 (6.3)*
Nursing home resident	78 (0.8)	79 (11.2)*	65 (18.9)	122 (21.6)	149 (7.8)	173 (4.7)*	113 (22.2)	74 (18.5)
**Nuvaxovid Regimen Type**								
Homologous	340 (10.0)	29 (4.2)*	12 (3.5)	<10 (< 1.0)*	108 (6.5)	261 (10.8)*	<10 (<1.0)	< 10 (< 1.0)
Heterologous	3061 (90.0)	661 (95.8)*	330 (96.5)	564 (99.8)*	1567 (93.6)	2155 (89.2)*	502 (98.8)	392 (98.3)
**Nuvaxovid Schedule**								
Primary series	1526 (31.2)	17 (2.4)*	<10 (<1.0)	0 (0.0)	246 (12.8)	1297 (35.2)*	<10 (<1.0)	< 10 (< 1.0)
Booster	3401 (69.5)	690 (97.7)*	342 (99.4)	565 (100)	1675 (87.4)	2416 (65.6)*	508 (99.8)	399 (99.8)
**Current COVID-19 Vaccine Dose** [a]								
<4	2430 (49.6)	90 (12.7)*	20 (5.8)	< 10 (< 1.0)*	534 (27.9)	1986 (53.9)*	10 (2.0)	16 (4.0)
≥ 4	2465 (50.4)	616 (87.3)*	324 (94.2)	559 (98.9)*	1383 (72.1)	1698 (46.1)*	499 (98.0)	384 (96.0)
**Use of Influenza Vaccine**								
Concomitant with Nuvaxovid	151 (3.1)	40 (5.7)*	NA	NA	90 (4.7)	101 (2.7)*	NA	NA

* *p*-value <0.05 subgroup differences, [a] For primary series, two doses are considered as one, CTV: Technical Committee on Vaccinations, NA: not applicable, SD: Standard deviation.

Supplemental Figure S1. Distribution of Nuvaxovid Recipients by CTV Comorbidities.

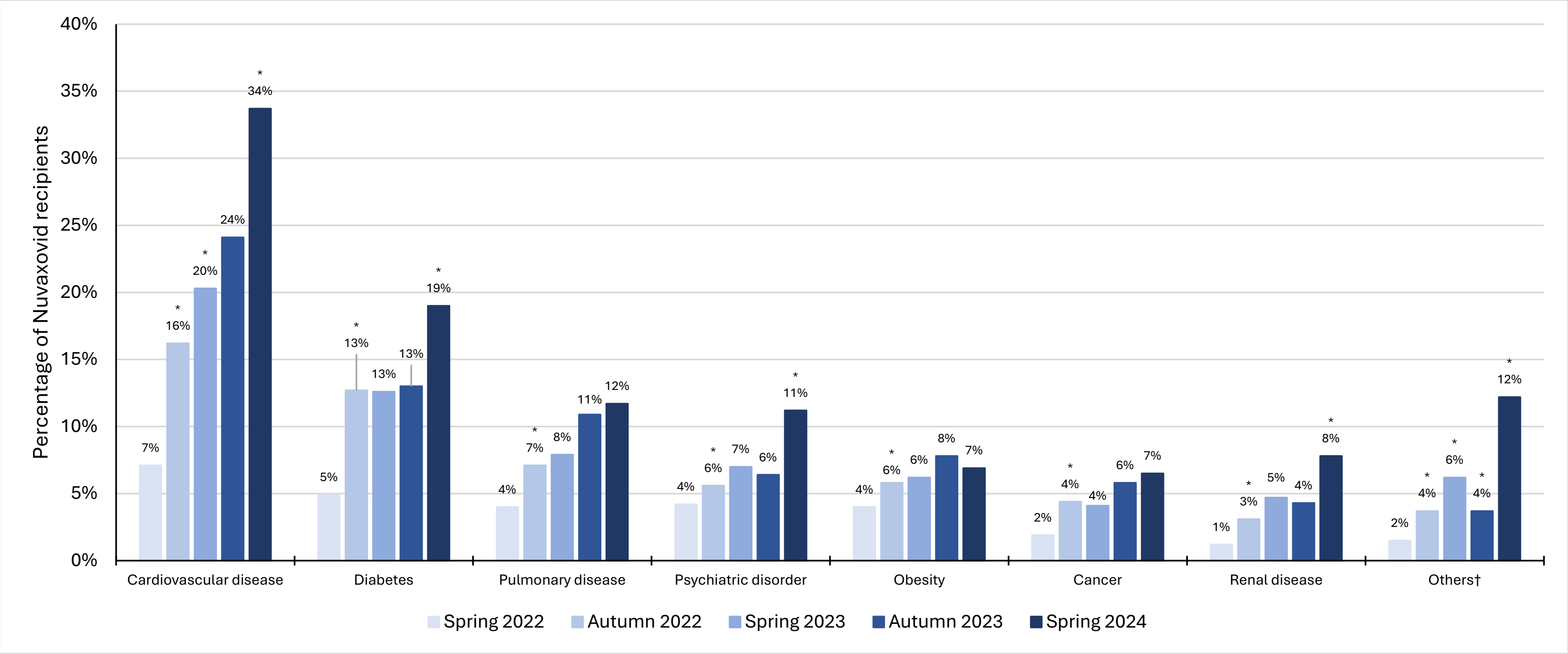

* *p*-value <0.05 versus previous season; † Sum of transplantation, trisomy 21, dementia, complicated hypertension, hepatic disease, CTV: Technical Committee on Vaccinations.
